# Well-Placed Acetabular Component Oriented Outside the Safe Zone During Weight-Bearing Daily Activities

**DOI:** 10.3389/fbioe.2021.664907

**Published:** 2021-06-10

**Authors:** Nan Zheng, Xiangjun Hu, Dimitris Dimitriou, Kerong Dai, Tao Guo, Tsung-Yuan Tsai

**Affiliations:** ^1^Med-X Research Institute, School of Biomedical Engineering, Shanghai Jiao Tong University, Shanghai, China; ^2^Engineering Research Center of Digital Medicine and Clinical Translation, Ministry of Education, Shanghai Jiao Tong University, Shanghai, China; ^3^Shanghai Key Laboratory of Orthopaedic Implants and Clinical Translation R&D Center of 3D Printing Technology, Department of Orthopaedic Surgery, Shanghai Ninth People’s Hospital, Shanghai Jiao Tong University School of Medicine, Shanghai, China; ^4^Department of Orthopaedics Bürgerspital Solothurn, Solothurn, Switzerland; ^5^Department of Orthopaedics, Guizhou Provincial People’s Hospital, Guiyang, China

**Keywords:** fluoroscopy, 2D-to-3D registration, total hip arthroplasty, acetabular cup orientation, *in vivo*, biomechanics

## Abstract

**Background:** A comprehensive and thorough understanding of functional acetabular component orientation is essential for optimizing the clinical outcome after total hip arthroplasty (THA). This study aimed to quantify the functional acetabular anteversion and inclination of unilateral THA patients during walking and static standing and to determine whether the functional acetabular orientation falls within the Lewinnek safe zone.

**Methods:** Seventeen patients with unilateral THA received a CT scan and dual fluoroscopic imaging during level walking and static standing to evaluate *in vivo* hip kinematics. The pelvic functional coordinate system of the 3D CT-based computer model was defined by the line of gravity and anterior pelvic plane (APP) to measure functional acetabular anteversion and inclination in different postures. The Lewinnek safe zone was used to determine the acetabular malposition during functional activities.

**Results:** The THA side demonstrated an average of 10.1° (± 9.6°, range –7.5° to 29.9°) larger functional anteversion and 16.0° (± 9.2°, range –7.2° to 29.9°) smaller inclination than native hips during level walking. Functional acetabular anteversion in the THA side during level walking and static standing was significantly larger than anatomical measurements (*p* < 0.05). Acetabular orientation of most well-placed THA components anatomically in the Lewinnek safe zone fell outside the safe zone during more than half of the gait cycle and static standing.

**Conclusion:** The current study revealed that an anatomically well-placed acetabular cup does not guarantee a well-functional orientation during daily activities. The *in vivo* mechanical performance and loading conditions of the THA component during other weight-bearing activities should be investigated in further studies.

## Introduction

Total hip arthroplasty (THA) is recognized as an effective surgical treatment for end-stage hip osteoarthritis, avascular necrosis, and other severe hip diseases to eliminate pain and restore hip function (Ethgen et al., 2004). Acetabular component malposition or malalignment in THA patients is a predictive risk factor for postoperative complications, such as dislocation (Jolles et al., 2002), impingement (Aamer et al., 2007), increased polyethylene liner wear and fracture, pelvic osteolysis (Kennedy et al., 1998; Little et al., 2009), and edge loading (Kwon et al., 2012). These complications are associated with a high revision rate after primary THA (Bozic et al., 2009). Lewinnek et al. (1978) first proposed the concept of “safe zone” as a reference to determine the ideal anteversion and inclination of the THA component in clinical practice. Yet recent studies (Elkins et al., 2015; Delsole et al., 2017; Murphy et al., 2018) reported that the acetabular component placed in the Lewinnek safe zone could not eliminate the risk of mechanical complications, including dislocation. Several investigators (McCollum and Gray, 1990; Callanan et al., 2011; Harrison et al., 2014; Murphy et al., 2018) suggested respective target zones with different shapes and ranges based on anatomical acetabular orientation measurements as safety guidelines to reduce the incidence of complications. However, there is no widely accepted target zone except the Lewinnek safe zone for THA component placement.

Previous studies primarily focused on anatomical acetabular anteversion and inclination using plain radiographs and compared these measurements in several static positions, including supine, sitting, and standing postures (Lewinnek et al., 1978; Lembeck et al., 2009; Lazennec et al., 2011; Tiberi et al., 2015; Pierrepont et al., 2017). The anterior pelvic plane (APP) is considered an anatomical reference plane to locate the component position (Babisch et al., 2008; Tsai et al., 2014). During activities, however, the movement of APP is associated with spinopelvic motion (Healy and William, 2009). The APP moves with the pelvis tilting posteriorly during sitting or supine to the standing position, resulting in significantly increased anteversion and inclination (Lembeck et al., 2009; Tiberi et al., 2015; Pierrepont et al., 2017). Therefore, if we measure the acetabular orientation using an anteroposterior (AP) radiograph, an anatomically well-placed acetabular component may become outside the safe zone in daily activities (Kwon et al., 2012; Tiberi et al., 2015; Heckmann et al., 2018; Tezuka et al., 2019). The spinopelvic motion contributes to abnormal mechanical loading conditions for THA during postural changes, including edge loading (Kwon et al., 2012) and impingement (Hara et al., 2016). The functional acetabular orientation associated with spinopelvic mobility may account for the risk of dislocation during activities for a well-placed acetabular component. Thus, a comprehensive and thorough understanding of functional acetabular component orientation is essential for optimizing the clinical outcome after THA. It is critical to evaluate the dynamic functional acetabular position during weight-bearing activities, such as level walking and static standing. Nevertheless, *in vivo* dynamic functional acetabular cup orientation in THA patients during walking remains unknown.

The purposes of the current study were to (1) quantify the functional acetabular anteversion and inclination during walking and static standing, (2) analyze the differences between functional and anatomical acetabular orientation, and (3) determine whether the functional acetabular orientation falls within the Lewinnek safe zone.

## Materials and Methods

### Patient Cohort

Our institutional review board approved this study. Seventeen patients (four males and 13 females) with primary unilateral THA were recruited, who provided written informed consent before participation. All recruited patients were diagnosed with unilateral end-stage osteoarthritis in hip joint and aged from 18 to 80 years. The gender of patients was not limited in the current study. All patients were implanted with cementless unilateral metal-on-polyethylene THA using the posterior approach. Their average age was 60.8 (± 8.6, range 47–73) years; average height and weight were 166.1 cm (± 10.6, range 137.2–180.3) and 76.3 kg (± 18.1, range 54.4–111.1), respectively; and body mass index (BMI) was 27.7 (± 6.4, range 19.7–43.4). The average follow-up period was 11.5 months (± 4.2, range 6.8–22.6). Patients with a history of any surgical complications or musculoskeletal injuries were excluded.

### CT-Based 3D Reconstruction and Anatomical Parameter Measurement

**FIGURE 1:**
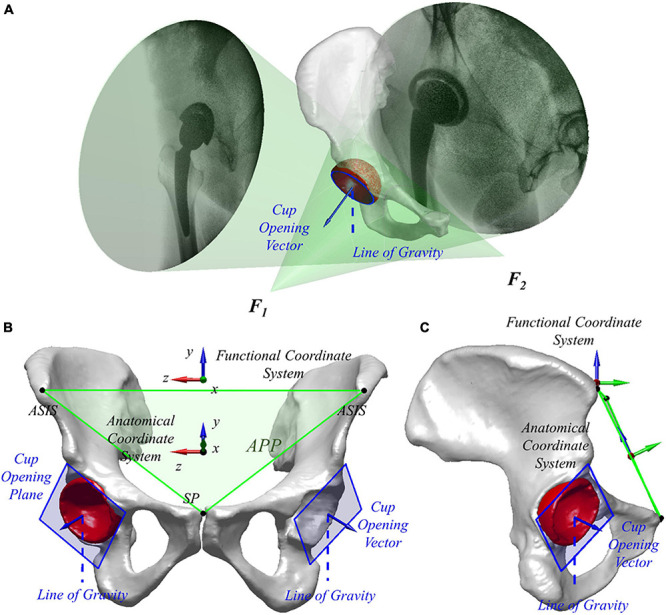
**(A)** Virtual environment of dual fluoroscopic imaging system (DFIS) with the line of gravity is shown. **(B,C)** Three-dimensional surface model of the pelvis and total hip arthroplasty (THA) acetabular implant in different views. The anterior pelvic plane (APP) and anatomical and functional coordinate systems are shown.

All patients received a 128-slice computed tomography (CT) scan (SOMATOM Definition AS1, Siemens, Germany) from the fifth lumbar vertebra to the proximal femur in a supine position. The CT images were segmented using a region-growing method in Amira 6.7.0 (Amira, Thermo Fisher Scientific, Rockford IL, United States) to create the 3D surface models of hip bone formed by ilium, ischium, and pubic bone of both THA and contralateral native sides as well as models of the acetabular cup in the implanted side. To eliminate the effects of pelvic tilt on the acetabular cup position, APP, established by the anterior superior iliac spine (ASIS) and pubic symphysis, was used as the reference plane to evaluate anatomical acetabular orientation (Lewinnek et al., 1978). The medial–lateral axis of the pelvis was set by connecting the left and right ASIS (Figures 1B,C). Thirty landmarks evenly distributed on the acetabular cup’s rim were manually selected based on the 3D surface models of the implanted and native acetabular cup and then fitted with a 3D plane using the least square method to determine the cup opening plane (Tsai et al., 2014). The anatomical acetabular anteversion was measured as the angle between the cup plane and frontal plane, with anatomical inclination measured as the angle between the cup opening plane and the transverse plane (Omri et al., 2010; Tsai et al., 2014). Radiographic acetabular orientations were calculated and compared with previous literature (Murray, 1993).

### Dual Fluoroscopic Imaging System and Functional Acetabular Orientation

Two mobile fluoroscopes (BV Pulsera, Phillips Medical, United States) were set in a nearly orthogonal position to form a dual fluoroscopic imaging system (DFIS). Each patient performed level walking on a treadmill at self-selected speed under the surveillance of DFIS (30 snapshots per second with an 8-ms pulse width) for the implanted and contralateral native hips. Also, the quiet standing postures of both hips of all the patients were imaged for comparison of the acetabular orientation in different functional poses. Next, the series of fluoroscopic images was imported into a virtual DFIS environment reconstructed in a customized program (MATLAB, MathWorks, Natick, MA), and the image planes, as well as X-ray sources of two fluoroscopes, were defined through a calibration procedure. The 3D bone models were imported into the program and manipulated in 6 degrees of freedom until the models matched the fluoroscopic images. The *in vivo* spatial positions of the hip and THA during dynamic level walking and static standing were reproduced (Figure 1A). The DFIS tracking technique for the THA and native hip was evaluated in a previous study with 0.35 mm and 0.55° accuracy (Tsai et al., 2013).

The pelvic functional coordinate system was used to determine *in vivo* acetabular orientation in different functional activities, i.e., dynamic level walking and static standing, for each patient. The *Y*-axis of the pelvic functional coordinate system was parallel to the gravity line directing to the superior. The *Z*-axis was defined as the cross product of the anterior normal vector of APP and *Y*-axis, pointing to the right with *X*-axis directing anteriorly (Figures 1B,C). The functional frontal plane position (i.e., YOZ plane in the pelvic functional coordinate system) and transverse plane (i.e., XOZ plane in the pelvic functional coordinate system) relative to the virtual DFIS environment changed with the movement of the pelvis during functional activities. The functional acetabular anteversion was measured as the angle between the cup plane and the functional frontal plane, with functional inclination measured as the angle between the cup opening plane and the functional transverse plane (Omri et al., 2010; Tsai et al., 2014). Dynamic functional acetabular orientation was quantified during level walking, while the static functional acetabular orientation was defined at static standing. Pelvic tilt was defined as the angle in the pelvic functional sagittal plane between APP and pelvic functional coronal plane.

To explicate the differences of anteversion and inclination relative to functional and anatomical coordinate systems, acetabular orientation in static standing was compared with anatomical angles. Besides, the functional and anatomical acetabular orientations of both THA and native hips were compared with the Lewinnek safe zone to determine whether its orientations were within or not. Acetabular orientations were considered as an essential factor in edge loading during weight-bearing activities such as the stance phase of walking and stair climbing (Kwon et al., 2012). Thus, durations of the acetabular orientation within the Lewinnek safe zone during the stance phase of level walking were calculated for each patient.

### Statistical Analysis

All measured parameters were presented as average and standard deviation and tested using the Kolmogorov–Smirnov test for normality. The paired *t*-test was applied for the parameters when normality criteria were met. Otherwise, the Wilcoxon signed-rank test was applied. The differences between functional and anatomical acetabular orientation were calculated for each patient, and Wilcoxon signed-rank tests were performed to determine whether the differences were statistically meaningful. The level of significance was set as 0.05.

## Results

### Functional Acetabular Anteversion and Inclination During Walking

**FIGURE 2:**
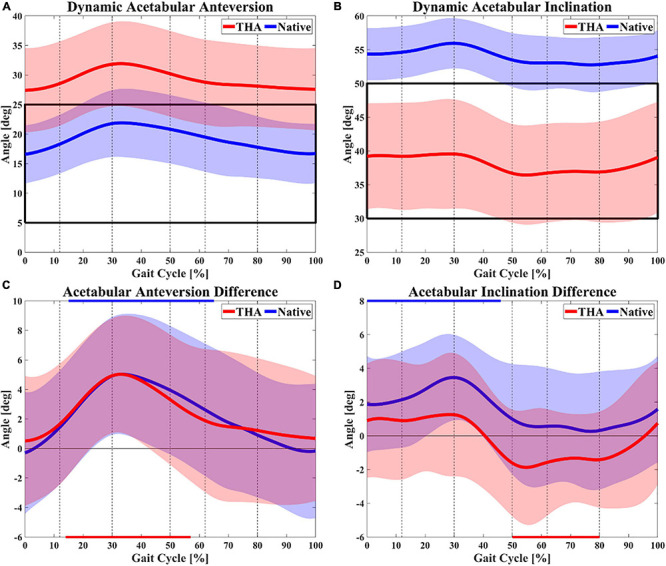
Dynamic functional acetabular anteversion **(A)** and inclination **(B)** of both total hip arthroplasty (THA) and native hips during gait. The black box marks the Lewinnek safe zone of 15 ± 10° for anteversion and 40 ± 10° for inclination. Differences between dynamic functional (gait) and anatomical anteversion **(C)** as well as inclination **(D)** are also shown (walking angle–anatomical angle). The red and blue lines on the horizontal axes indicate whether there was significant difference between walking and anatomical angle for THA and native hips, respectively. The dotted lines divide gait cycle into loading response (0∼12%), midstance (12∼30%), terminal stance (30∼50%), pre-swing (50∼62%), acceleration swing (62∼80%), and deceleration swing (80∼100%).

On the THA side, the functional anteversion of the acetabular component was positive from the loading response to the midstance of the gait cycle and reached the peak anteversion of 31.9 ± 7.2° at 33% of the gait cycle. After that, the acetabular anteversion kept decreasing until the next heel strike (Figure 2A). The average range and angle of functional anteversion for THA during gait were 5.3 ± 1.5 and 29.3 ± 7.0°, respectively. A similar pattern of dynamic functional anteversion was observed in the contralateral native side with anteversion increasing to 21.9 ± 5.8° during 0∼33% of the gait cycle and decreasing during 33∼100% of the gait cycle (Figure 2A). The average range and angle of functional anteversion for native hips during gait were 6.4 ± 1.4 and 19.2 ± 5.3°, respectively. The functional anteversion of THAs was 10.1 ± 9.6° larger than native hips on average during level walking, with a wide range of –7.5–29.9°.

The functional inclination of the acetabular component in the THA side increased to 39.6 ± 8.1° through 0∼29% of the gait cycle, decreased to 36.4 ± 7.3° from the terminal stance to pre-swing (55%), and then increased slightly to 39.0 ± 8.2° in the swing phase (Figure 2B). The average range and angle of functional inclination for THA during gait were 5.9 ± 2.2 and 38.1 ± 7.5°, respectively. The motion of functional inclination in the native side showed a similar pattern to the THA side. For native hips, functional inclination demonstrated an increasing trend to 55.9 ± 3.7° from heel strike to midstance (30%) and a decreasing trend to 53.0 ± 4.0° through terminal stance and pre-swing (59%). In contrast to the THA side, the functional inclination of native hips remained nearly constant during the acceleration swing and increased again until the next heel strike (Figure 2B). The average range and angle of functional inclination for native hips during gait were 5.0 ± 2.1 and 54.0 ± 3.6°, respectively. The functional inclination of THAs was 16.0 ± 9.6° smaller than native hips on average during level walking, with a wide range of –7.2–29.9°. On average, THAs had significantly greater functional anteversion (*p* < 0.01) with less functional inclination (*p* < 0.01) than native hips during level walking. The average pelvic tilt was –3.2 ± 0.8° in the THA side (range 2.7°, from –4.7 to –2.0°) and –2.8 ± 1.6° in the native hip side (range 2.0°, from –3.6 to –1.6°) during level walking.

### Differences Between Functional and Anatomical Acetabular Orientation

Significant differences between dynamic functional acetabular orientation during gait and anatomical angles were reported (Table 1). Compared with anatomical measurements, the functional anteversion of THA significantly increased during 14∼57% of the gait cycle (*p* < 0.05) with a maximal increase of 5.0 ± 4.0° at 33% of the gait cycle (*p* < 0.001). The functional anteversion in contralateral native hips significantly increased throughout most of the stance phase (15∼65%, *p* < 0.05), with a maximal increase of 5.0 ± 4.0° at 33% of the gait cycle (*p* < 0.001; Figure 2C). On average, the acetabular cups were 2.4 ± 4.6° anteverted for THAs and 2.3 ± 4.5° anteverted for native hips during functional level walking compared with the anatomical position (Table 2). The dynamic functional inclination of THAs was less than anatomical angle through nearly half of the gait cycle (41∼95%), and the difference was statistically significant during the 50∼80% (*p* < 0.05) with the maximal decrease of 1.9 ± 3.4° at 55% of the gait cycle (*p* < 0.05; Figure 2D). Functional inclination was 1.2 ± 3.7° greater than anatomical angle when it reached the peak at 29% of the gait cycle (*p* = 0.16), and the average difference between functional and anatomical inclination for THA was –0.3 ± 3.4° (Table 2). Besides, native hips had greater functional inclination than anatomical angles during the entire gait cycle. Statistically significant differences were observed from heel strike to terminal stance (0∼46%, *p* < 0.05) with a maximal increase of 3.5 ± 2.5° at 30% of the gait cycle (*p* < 0.001; Figure 2D). Functional inclination in the native side had a 1.5 ± 3.2° increase compared with anatomical angle on average, which was greater than the THA side (Table 2). Functional anteversion in standing position was 5.9 ± 5.3° significantly greater than anatomical orientation in THAs (*p* < 0.05) and 4.9 ± 4.6° significantly greater in native hips (*p* < 0.05), as well as functional inclination, 2.2 ± 3.1° greater in THAs (*p* = 0.40) and 3.0 ± 4.3° significantly greater in native hips (*p* < 0.05; Table 2).

**TABLE 1:** The anatomical acetabular orientation and functional acetabular orientation at standing (static) position and during level walking (dynamic) were calculated in both THA and native hips of unilateral THA patients.

**Subject ID**	**THA**						**Native hip**					
	**Anteversion (°)**			**Inclination (°)**			**Anteversion (°)**			**Inclination (°)**		
	**Walking**	**Standing**	**Anatomical**	**Walking**	**Standing**	**Anatomical**	**Walking**	**Standing**	**Anatomical**	**Walking**	**Standing**	**Anatomical**
1	18.2 (2.3)	25.1	18.6	40.2 (3.1)	43.9	39.6	13.3 (2.3)	18.9	12.5	52.6 (3.4)	53.0	53.4
2	26.5 (2.2)	31.5	28.6	41.2 (1.2)	45.4	41.3	34.0 (3.3)	37.1	31.8	58.9 (1.9)	61.2	58.5
3	25.5 (1.6)	29.6	22.3	40.7 (0.8)	40.7	38.2	22.7 (1.7)	24.2	16.6	53.6 (1.1)	57.7	51.5
4	31.5 (0.8)	33.6	33.1	29.8 (1.5)	31.1	30.7	16.5 (2.1)	19.4	17.5	52.2 (1.4)	57.3	53.0
5	35.0 (1.8)	42.1	35.4	42.4 (1.7)	48.5	39.9	18.6 (2.1)	24.2	19.9	48.1 (2.1)	49.2	53.1
6	20.1 (2.2)	40.4	21.6	26.9 (1.2)	35.1	31.5	18.1 (2.1)	30.6	20.2	53.4 (1.1)	63.3	50.2
7	37.1 (1.6)	38.9	39.5	37.0 (1.4)	40.9	44.8	10.4 (1.9)	10.6	15.1	61.4 (1.3)	58.0	58.3
8	27.9 (1.3)	28.6	20.2	31.3 (1.2)	31.0	31.4	19.4 (2.3)	18.5	10.3	53.5 (0.9)	54.4	48.8
9	25.4 (1.5)	27.9	19.1	29.1 (2.5)	29.0	31.4	16.8 (0.8)	20.3	12.0	59.0 (1.4)	62.9	54.7
10	34.2 (2.0)	36.5	23.4	34.7 (2.9)	37.1	32.1	19.5 (2.0)	22.4	11.7	57.6 (2.1)	60.1	53.4
11	31.9 (1.9)	30.6	30.2	40.9 (2.1)	41.6	43.0	14.6 (1.5)	13.7	16.7	48.8 (1.2)	48.5	46.6
12	46.4 (2.6)	50.2	51.0	46.3 (2.8)	52.9	48.6	16.5 (2.7)	18.7	16.8	53.2 (1.9)	53.8	56.4
13	23.3 (2.2)	28.5	17.5	36.0 (1.4)	39.2	36.1	22.6 (1.8)	25.5	17.2	50.0 (1.8)	52.0	46.8
14	36.1 (1.2)	35.8	34.6	59.1 (1.3)	59.1	56.8	22.1 (2.5)	19.6	20.6	51.9 (1.4)	50.0	51.7
15	26.2 (2.0)	25.0	18.9	34.5 (1.5)	34.6	32.2	23.5 (2.7)	21.4	15.6	56.2 (1.0)	56.1	53.9
16	26.1 (0.9)	25.3	20.8	38.4 (1.4)	37.7	37.6	15.5 (2.3)	22.1	12.7	53.7 (1.1)	51.8	49.1
17	25.7 (1.5)	28.8	22.5	38.4 (2.8)	40.8	36.1	22.1 (1.9)	23.5	19.6	54.3 (0.9)	53.4	53.2
Average	29.3 (7.0)	32.9 (7.0)	26.9 (9.3)	38.1 (7.5)	40.5 (7.9)	38.3 (7.1)	19.2 (5.3)	21.8 (6.0)	16.9 (5.0)	54.0 (3.6)	55.5 (4.6)	52.5 (3.5)

**THA, total hip arthroplasty.**

**TABLE 2:** Differences between functional and anatomical acetabular anteversion and inclination in both THA and native hips for unilateral THA patients.

**Subject ID**	**THA**				**Native hip**			
	**Anteversion (°)**		**Inclination (°)**		**Anteversion (°)**		**Inclination (°)**	
	**Walking-anatomical**	**Standing-anatomical**	**Walking-anatomical**	**Standing-anatomical**	**Walking-anatomical**	**Standing-anatomical**	**Walking-anatomical**	**Standing-anatomical**
1	–0.3 (2.3)	6.6	0.7 (3.1)	4.4	0.8 (2.3)	6.3	–0.8 (3.4)	–0.4
2	–2.1 (2.2)	2.9	–0.1 (1.2)	4.1	2.2 (3.3)	5.3	0.4 (1.9)	2.7
3	3.2 (1.6)	7.2	2.5 (0.8)	2.5	6.1 (1.7)	7.6	2.1 (1.1)	6.2
4	–1.6 (0.8)	0.4	–0.9 (1.5)	0.4	–1.0 (2.1)	1.9	–0.8 (1.4)	4.4
5	–0.4 (1.8)	6.7	2.5 (1.7)	8.6	–1.3 (2.1)	4.3	–5.0 (2.1)	–3.8
6	–1.5 (2.2)	18.9	–4.6 (1.2)	3.6	–2.0 (2.1)	10.4	3.2 (1.1)	13.2
7	–2.4 (1.6)	–0.6	–7.8 (1.4)	–3.9	–4.7 (1.9)	–4.5	3.2 (1.3)	–0.2
8	7.7 (1.3)	8.4	–0.1 (1.2)	–0.4	9.1 (2.3)	8.2	4.7 (0.9)	5.5
9	6.3 (1.5)	8.7	–2.2 (2.5)	–2.4	4.8 (0.8)	8.3	4.4 (1.4)	8.2
10	10.8 (2)	13.1	2.6 (2.9)	5.0	7.7 (2.0)	10.7	4.2 (2.1)	6.6
11	1.7 (1.9)	0.4	–2.1 (2.1)	–1.4	–2.1 (1.5)	–3.0	2.1 (1.2)	1.9
12	–4.6 (2.6)	–0.8	–2.3 (2.8)	4.4	–0.3 (2.7)	1.9	–3.2 (1.9)	–2.5
13	5.9 (2.2)	11.0	–0.1 (1.4)	3.1	5.3 (1.8)	8.2	3.2 (1.8)	5.2
14	1.5 (1.2)	1.2	2.3 (1.3)	2.3	1.5 (2.5)	–1.0	0.3 (1.4)	–1.7
15	7.3 (2.0)	6.1	2.3 (1.5)	2.4	7.9 (2.7)	5.8	2.3 (1.0)	2.2
16	5.3 (0.9)	4.5	0.8 (1.4)	0.1	2.8 (2.3)	9.4	4.7 (1.1)	2.8
17	3.3 (1.5)	6.3	2.3 (2.8)	4.7	2.5 (1.9)	3.9	1.1 (0.9)	0.2
Average	2.4 (4.6)	5.9 (5.3)	–0.3 (3.4)	2.2 (3.1)	2.3 (4.5)	4.9 (4.6)	1.5 (3.2)	3.0 (4.3)

**THA, total hip arthroplasty.**

### Acetabular Orientation Compared With the Lewinnek Safe Zone

**FIGURE 3:**
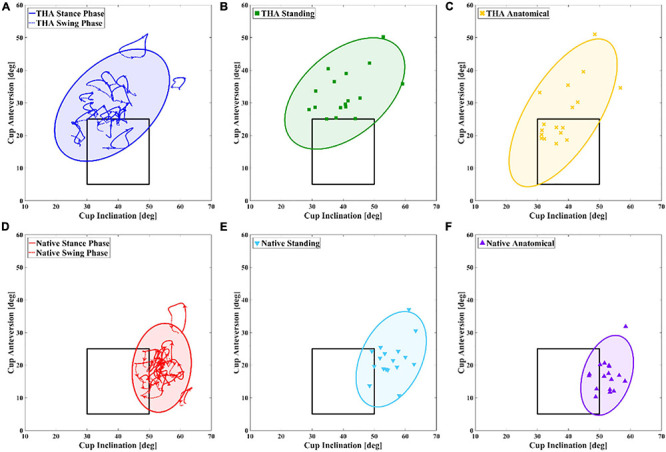
Distributions of different acetabular orientations in total hip arthroplasty (THA) **(A–C)** and native hip side **(D–F)** for each unilateral THA patient are shown. Dynamic functional angles in level walking, static functional angles in standing position, and anatomical angles are shown. The blue, green, and yellow 95% group ellipses represent distribution of measurements using different definitions in the THA side, while red, light blue, and violet 95% group ellipses represent distribution of measurements using different definitions in the contralateral native side.

The functional acetabular orientation was compared with the Lewinnek safe zone to determine whether malposition occurred in functional activities, and significant individual variations in functional and anatomical acetabular orientation were noted (Figure 3). Ten of 17 (58.8%) THAs had anatomical acetabular orientation within the safe zone. However, only two of these 10 THAs had normal dynamic functional angles over 60% duration of the stance phase, with the functional angle of other eight THAs inside the safe zone in less than 25% of the stance phase. On average, these anatomically well-placed THAs had functional acetabular orientation within the safe zone only in 23.0% duration of the stance phase and 44.4% duration of the swing phase. One of seven THAs with abnormal anatomical orientation had acetabular orientation in the safe zone during 6.3% of the stance phase and 66.7% of the swing phase, while the acetabular orientation of the other six THAs fell outside the safe zone during the entire gait cycle (Table 3 and Figure 3A). The static functional acetabular orientation of THAs demonstrated that all 17 THAs had greater anteversion than 25° in static standing whether anatomical angles of THAs were within the safe zone or not (Tables 1, 3 and Figure 3B). The anatomical acetabular orientation of 4/17 native hips (23.5%) was within the safe zone. Only one of these four hips kept inside the safe zone during 74.6% of the stance phase and 87.2% of the swing phase (Table 3 and Figure 3D). On average, these four native hips with normal anatomical angles had acetabular orientation in the safe zone in 25.0% of the stance phase and 46.8% duration of the swing phase. Besides, one native hip with normal anatomical angle and two native hips with abnormal anatomical angle had acetabular orientation inside the safe zone in static standing (Tables 1, 3 and Figure 3E).

**TABLE 3:** Duration of stance phase and gait cycle when THAs and native hips stayed in the Lewinnek safe zone were calculated for each individual patient.

**Subject ID**	**THA**				**Native hip**			
	**Walking**		**Standing**	**Anatomical**	**Walking**		**Standing**	**Anatomical**
	**Stance (%)**	**Swing (%)**			**Stance (%)**	**Swing (%)**		
1	100	100		Y		59		
2	6.3	66.7						
3	15.9	100		Y				
4					7.9			
5					63.5	100	Y	
6				Y				
7								
8				Y				Y
9	1.6			Y				
10				Y				
11					74.6	87.2	Y	Y
12					1.6			
13	66.7	84.6		Y	25.4	100		Y
14						33.3	Y	
15	6.3	79.5		Y				
16	19.0			Y				Y
17	20.6	79.5		Y				

*Whether the anatomical and functional (standing) acetabular orientations lie within the Lewinnek safe zone for each patient were also noted. Y indicates anatomical or functional (standing) acetabular orientation is within the Lewinnek safe zone. THA, total hip arthroplasty.*

## Discussion

The current study quantified *in vivo* functional acetabular orientation in patients following unilateral THA during level walking and static standing. Significantly greater functional anteversion and less inclination were observed in THAs during level walking when compared with native hips. The functional acetabular anteversions and inclinations of both THAs and native hips in walking and static standing were larger than anatomical angles, except for inclination of THAs during gait. Besides, the functional anteversion in THA, as well as inclination in native hips, was located outside the Lewinnek safe zone in the entire gait cycle. There were notable individual variations in functional and anatomical acetabular orientations. THAs with normal anatomical orientation had functional orientation outside the Lewinnek safe zone during level walking and static standing.

No previous study evaluated the relationship between pelvic tilt and acetabular orientation in continuous weight-bearing activities. Lembeck et al. (2009) quantified the non-linear relationship between pelvic tilt and angles of acetabular anteversion and inclination using trigonometric functions, indicating that the pelvic tilt of 1° will change the anteversion of 0.7° and inclination of 0.3°. Other researchers (Lazennec et al., 2011; Stefl et al., 2017) reported that increased pelvic tilt in supine to standing and standing to sitting positions was highly associated with a decrease of anteversion and inclination. A similar tendency was observed in the current study, demonstrating that an increase in the pelvic tilt of 1° decreased functional anteversion of 1.7° in THAs and 2.5° in native hips, as well as the inclination of 1.1° in THAs and 1.6° in native hips. The magnitude of functional anteversion and inclination changes relative to pelvic tilt in this study was more significant than in previous studies (Lembeck et al., 2009; Lazennec et al., 2011; Stefl et al., 2017), and the underestimated effect of pelvic tilt on acetabular orientation emphasized the evaluation of pelvic motion during activities in preoperative planning. Furthermore, acetabular anteversion and inclination in static standing were higher than measurements during level walking for most individual patients due to the trunk and pelvis tilting forward in standing posture (Table 1).

There were significant differences in functional acetabular orientation between THAs and native hips during both level walking and static standing activities. THA cannot completely restore normal hip anatomy compared with non-implanted native hip (Tsai et al., 2014), and asymmetric hip joint kinematics following THA were observed in walking and stair-climbing activities (Dimitris et al., 2015; Tsai et al., 2015), indicating that common differences between THAs and native hips in cup position and hip movement exist for patients undergoing unilateral THA. In the current study, we observed an average greater functional anteversion and smaller inclination of THAs than native hips in weight-bearing level walking and static standing (Table 1). Less increase of functional inclination compared with anatomical angle in THAs than native hips during the double-leg stance of the gait cycle was also noted (Figure 2D). The pelvic drop and hip abduction in the THA side were considered as compensation for abductor weakness and upper limb inclination during gait (Baker and Bitounis, 1989; Madsen et al., 2004; Nantel et al., 2009; Zhou et al., 2012), and the asymmetric hip kinematics and muscle contracture may account for the differences of functional acetabular anteversion and inclination between THA and native hips in weight-bearing activities (Madsen et al., 2004; Dimitris et al., 2015). The differences of measured parameters reported in this study indicated that these THAs did not fully restore native hip function during gait around 1-year after surgery. Also, the distributions of measured functional and anatomical angles for THAs and native hips were reported (Figure 3), suggesting that individual variation of both functional and anatomical measurements for THAs was larger than native hips. It may result from specific pelvic anatomy, cup placement, and personal walking pattern for each patient (Zhou et al., 2012; Seagrave et al., 2017).

**FIGURE 4:**
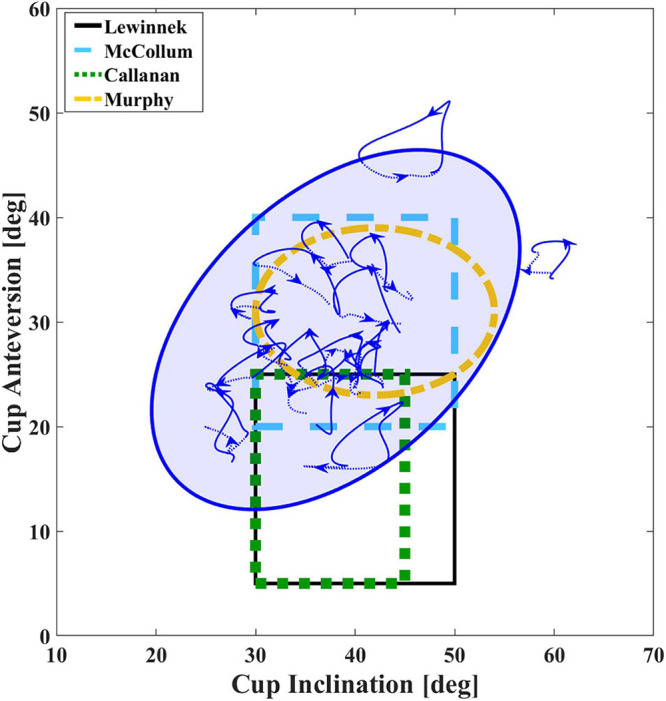
Average dynamic functional acetabular anteversion and inclination of total hip arthroplasty (THA) during gait. Different definitions of safe zones are shown (Lewinnek et al., 1978; McCollum and Gray, 1990; Callanan et al., 2011; Murphy et al., 2018). The blue 95% group ellipse represents distribution of functional acetabular orientation during gait for the THA side.

Lewinnek et al. (1978) first suggested the “safe zone” concept for sufficient cup coverage and hip mobility following THA. However, there was no significant difference in the dislocation rate of whether the acetabular component was placed in the Lewinnek safe zone or not (Dorr and Callaghan, 2019), highlighting the importance of functional stability in daily activities. Several factors may affect the functional stability of THA, including cup coverage (Knight and Atwater, 1992), spinopelvic imbalance (Tezuka et al., 2019), and poor abductor function (Dimitris et al., 2015). Tezuka et al. (2019) introduced a functional safe zone for clinical practice based on the motion of spinopelvic and hip joints in standing and sitting positions rather than considering the static component position merely. In the current study, anatomical measurements of 10/17 (58.8%) THAs were within the Lewinnek safe zone, while these 10 THAs had normal acetabular orientation within the safe zone only in 23.0% duration of the stance phase (Table 3), suggesting that the well-placed acetabular component in surgery had functional acetabular orientation outside the Lewinnek safe zone during the most duration of gait. The absence of functional evaluation for the *in vivo* acetabular position may contribute to the poor predictive value of the traditional the Lewinnek safe zone based on anatomical measurement (Elkins et al., 2015; Dorr and Callaghan, 2019). The functional acetabular orientation during gait in the current study was also compared with several proposed target zones (McCollum and Gray, 1990; Delsole et al., 2017; Murphy et al., 2018), and the wide-range distribution of measured parameters indicated that the target zone with larger anteversion might be more rational in functional acetabular evaluation in level walking (Figure 4). In addition, we observed similar functional acetabular orientation in level walking and static standing on average. However, the pattern of acetabular orientation with a specific range for each patient was unique (Figure 3A), which was different from the measures in standing. The change of anteversion and inclination relative to the gravity line during the gait cycle would associate with different mechanical loading conditions (Kwon et al., 2012; Hara et al., 2016). Recent numerical studies have demonstrated that different combinations of the range of hip joint motion, prosthesis design and implant parameters, and acetabular orientation lead to the different risks of impingement after THA surgery (Putame et al., 2019; Widmer, 2020). These may account for the dislocations of well-placed THAs in weight-bearing daily activities. Thus, we suggest taking functional acetabular orientation into account in pre-surgery planning and postoperative evaluation, and *in vivo* performance of THA component in further activities including sitting and stair climbing should be investigated.

Several limitations of the current study should be noted. First, no poor functioning patients suffering from dislocation were recruited. There was a lack of long-term follow-up data to explain the relationship between functional acetabular orientation and complication rate. Thus, a safe zone during level walking or static standing can hardly be proposed. However, the current study provided valuable information for functional acetabular evaluation during weight-bearing activities. Finally, several factors causing postoperative complications, such as age, surgical approach, femoral offset, bearing type, and spinopelvic mobility (Seagrave et al., 2017), were not investigated in the current study due to the small number of patients.

## Conclusion

In conclusion, the current study first quantified continuous acetabular orientation during weight-bearing functional activities. The larger functional anteversion and smaller inclination were observed in THAs compared with native hips during level walking. THAs demonstrated a range of 5.3 ± 1.5° of anteversion and 5.9 ± 2.2° of inclination, while native hips had a range of 6.4 ± 1.4° of anteversion and 5.0 ± 2.1° of inclination. Functional acetabular anteversion and inclination measured in both level walking and static standing were greater than anatomical angles, and component anatomically placed in the Lewinnek safe zone had functional acetabular orientation outside the safe zone in the most duration of the gait cycle and static standing. The current study suggested taking the functional acetabular into account in pre-surgery planning and postoperative evaluation. The *in vivo* mechanical performance and loading conditions of THA components during other weight-bearing activities should be investigated in further studies.

## Data Availability Statement

The raw data supporting the conclusions of this article will be made available by the authors, without undue reservation.

## Ethics Statement

The protocol was approved by the Ethics Committee of Shanghai Sixth People’s Hospital, China (No. 2019026). Written informed consent was obtained from all patients enrolled in the investigation. The patients/participants provided their written informed consent to participate in this study.

## Author Contributions

NZ, XH, TG, and T-YT contributed to the conception, design, provision of study materials and patients, and collection and assembly of data. NZ, XH, and T-YT carried out the data analysis. NZ, XH, DD, KD, TG, and T-YT contributed to data interpretation. NZ, XH, DD, and T-YT participated in the writing of the manuscript. All authors gave approval of the final manuscript.

## Conflict of Interest

The authors declare that the research was conducted in the absence of any commercial or financial relationships that could be construed as a potential conflict of interest.
